# A Mutation Causes MuSK Reduced Sensitivity to Agrin and Congenital Myasthenia

**DOI:** 10.1371/journal.pone.0053826

**Published:** 2013-01-09

**Authors:** Asma Ben Ammar, Payam Soltanzadeh, Stéphanie Bauché, Pascale Richard, Evelyne Goillot, Ruth Herbst, Karen Gaudon, Caroline Huzé, Laurent Schaeffer, Yuji Yamanashi, Osamu Higuchi, Antoine Taly, Jeanine Koenig, Jean-Paul Leroy, Fayçal Hentati, Hossein Najmabadi, Kimia Kahrizi, Manouchehr Ilkhani, Michel Fardeau, Bruno Eymard, Daniel Hantaï

**Affiliations:** 1 Inserm, UMRS 975, UPMC, Institut du Cerveau et de la Moelle épinière, Groupe Hospitalier Pitié-Salpêtrière, Paris, France; 2 Laboratoire de Neurobiologie Moléculaire et Neuropathologie, Institut National de Neurologie, Université Tunis El Manar, La Rabta, Tunis, Tunisia; 3 Ecole Pratique des Hautes Etudes, Paris, France; 4 APHP, UF Cardiogénétique et Myogénétique, Service de Biochimie Métabolique, Groupe Hospitalier Pitié-Salpêtrière, Paris, France; 5 Equipe Différenciation Neuromusculaire, IFR128, UMR5161, ENS Lyon, CNRS, INRA, Université de Lyon, Lyon, France; 6 Medical University of Vienna, Center for Brain Research, Vienna, Austria; 7 Division of Genetics, Department of Cancer Biology, the Institute of Medical Science, the University of Tokyo, Tokyo, Japan; 8 Laboratoire de Conception et Application de Molécules Bioactives, UMR 7199 CNRS-Université de Strasbourg, Illkirch, France; 9 University of Social Welfare and Rehabilitation Sciences, Genetics Research Center, Tehran, Islamic Republic of Iran; 10 Shahid Beheshti University of Medical Sciences, Department of Neurology, Tehran, Islamic Republic of Iran; 11 Unité de Morphologie Neuromusculaire, Institut de Myologie, Groupe Hospitalier Pitié-Salpêtrière, Paris, France; 12 APHP, Centre de Référence en Pathologie Neuromusculaire Paris-Est, Institut de Myologie, Groupe Hospitalier Pitié-Salpêtrière, Paris, France; Weizmann Institute of Science, Israel

## Abstract

Congenital myasthenic syndromes (CMSs) are a heterogeneous group of genetic disorders affecting neuromuscular transmission. The agrin/muscle-specific kinase (MuSK) pathway is critical for proper development and maintenance of the neuromuscular junction (NMJ). We report here an Iranian patient in whom CMS was diagnosed since he presented with congenital and fluctuating bilateral symmetric ptosis, upward gaze palsy and slowly progressive muscle weakness leading to loss of ambulation. Genetic analysis of the patient revealed a homozygous missense mutation c.2503A>G in the coding sequence of *MUSK* leading to the p.Met835Val substitution. The mutation was inherited from the two parents who were heterozygous according to the notion of consanguinity. Immunocytochemical and electron microscopy studies of biopsied deltoid muscle showed dramatic changes in pre- and post-synaptic elements of the NMJs. These changes induced a process of denervation/reinnervation in native NMJs and the formation, by an adaptive mechanism, of newly formed and ectopic NMJs. Aberrant axonal outgrowth, decreased nerve terminal ramification and nodal axonal sprouting were also noted. *In vivo* electroporation of the mutated MuSK in a mouse model showed disorganized NMJs and aberrant axonal growth reproducing a phenotype similar to that observed in the patient’s biopsy specimen. *In vitro* experiments showed that the mutation alters agrin-dependent acetylcholine receptor aggregation, causes a constitutive activation of MuSK and a decrease in its agrin- and Dok-7-dependent phosphorylation.

## Introduction

The NMJ is a highly specialized structure formed by a motoneuron, a terminal Schwann cell and a muscle fiber. The contact between the nerve terminal and the muscle membrane constitutes a privileged zone in which neurotransmission occurs. At this synapse, the nerve terminal organizes postsynaptic differentiation by releasing a proteoglycan called agrin which binds to its receptor, a low-density lipoprotein receptor (LDLR)-related protein (Lrp4) located on the postsynaptic membrane [1]–[3]. Lrp4 forms a complex with the muscle-specific tyrosine kinase (MuSK) which plays a central role in the organization of the postsynaptic scaffold. MuSK activation is required to recruit downstream signaling components that trigger the local aggregation and synthesis of postsynaptic nicotinic acetylcholine receptors (nAChRs) and several other proteins, such as the cytoskeletal protein rapsyn [4]–[6].

MuSK is a tyrosine kinase receptor with an ectodomain containing three immunoglobulin (Ig)-like domains and a Frizzled-like cysteine-rich domain (initially described as a C box plus a fourth Ig-like domain), a transmembrane-spanning region, and the intracellular region including a juxtamembrane domain, a kinase domain, and a short C-terminal tail [7]–[9]. In general, ligand binding to the extracellular portion of tyrosine kinase receptors results in autophosphorylation of specific tyrosine residues, which in turn increases the catalytic activity of the receptor and creates binding sites for other signaling proteins. The kinase activation loop (so-called “A loop”), whose conformation is changed for substrate interaction, and the juxtamembrane region located between the transmembrane helix and the tyrosine kinase domain are important regulatory regions for MuSK kinase activity [10]–[12]. The juxtamembrane region of MuSK also contains a NPXY binding motif for the phosphotyrosine binding (PTB) domain of the cytoplasmic adapter-like protein Dok-7, which plays an essential role in the regulation of MuSK phosphorylation [13]–[15].

The critical role of MuSK signaling is supported by the fact that mice deficient in agrin, Lrp4, MuSK, or Dok-7 lack NMJs and die at birth from respiratory failure [14], [16], [17]. Consistently, RNA interference directed against MuSK or conditional postnatal inactivation of the *musk* gene causes disassembly of the postsynaptic components of NMJs [18], [19].

Congenital myasthenic syndromes (CMSs) are a heterogeneous group of genetic disorders that give rise to neuromuscular transmission defects. CMSs are conveniently classified according to their target as presynaptic, synaptic (basal lamina-associated) or postsynaptic [20]. Postsynaptic CMSs are currently the most common category of CMSs and are usually the consequence of mutations in genes encoding the four adult muscle nAChR subunits or rapsyn [21], [22]. It has recently been shown that mutations in genes encoding proteins involved in the critical signal transduction pathway of MuSK requiring neural agrin, the muscle cytoplasmic protein Dok-7 and MuSK itself can also result in severe forms of CMSs [23]–[25]. Since our first demonstration that mutations in MuSK underlie a CMS, two other reports implicating MuSK mutations have been published: a homozygous missense mutation in the ectodomain of MuSK [26] and heterozygous missense mutations in its kinase domain [27]. Here we describe and characterize a patient with a novel homozygous mutation in *MUSK* affecting the kinase domain.

## Materials and Methods

### Ethics Statement

Muscle biopsy and/or blood samples of patients were obtained after informed written consent by the parents for themselves and on behalf of their children was secured in accordance with the protocol approved by the ethics committee of La Salpêtrière Hospital (CCPPRB #93-02).

Experimental procedures on mice were performed in accordance with European legislation (L358-86/609/EEC) and approved locally by the Inserm.

### Histopathologic Studies of Patient’s Biopsied Muscle

Open biopsy was performed on the deltoid muscle. The NMJ-containing zone was determined by small twitches provoked by the scalpel tip and confirmed by the presence of cholinesterase activity using the classic technique of Koelle and Friedenwald [28]. Whole fibers fixed in 4% paraformaldehyde were stained for AChRs with TRITC-labeled alpha-bungarotoxin (α-BGT) (Molecular Probes, Leiden, The Netherlands), and for neurofilaments with an anti-168 kD neurofilament antibody (2H3 clone, Hybridoma Bank, Iowa City, IA) or an anti-200 kD neurofilament antibody (RT 97 clone, Boehringer Ingelheim, Reims, France). In addition, cryostat sections were labeled with α-BGT and an anti-MuSK antibody (kindly donated by Markus Rüegg, Basel, Switzerland). Both whole fibers and cryostat sections were observed with a confocal microscope (Zeiss, LSM 510) and images were acquired with the equivalent settings (laser power, gain, magnification, thickness of the stacks). The area of synaptic gutters per NMJ and the mean α-bungarotoxin fluorescence intensity within that area were measured using Metamorph software (Universal Imaging, Westchester, PA, USA). Fluorescence intensity quantification of MuSK was not feasible on whole mount preparation because of the lack of sample and was therefore performed on cryostat section using the same software. Electron microscopy of the NMJ was performed by conventional methods.

### Quantitative RT-PCR Analysis

Total RNA was extracted from 10 consecutive 10 µm cryostat sections of biopsied deltoid muscle using the Qiagen RNAeasy mini kit (Qiagen), with DNase treatments. QRT-PCR was performed according to the manufacturer’s instructions with the Quantitect one-step RT-PCR SYBRGreen DNA detection kit (Qiagen) in a Light-Cycler (Roche Diagnostics, France). Primer sequences were as follows. γ-subunit AChR: forward 5′-AACGAGACTCGGATGTGGTC-3′, reverse 5′-GTCGCACCACTGCATCTCTA-3′; actin: forward 5′-GGACTTCGAGCAAGAGATGG-3′, reverse 5′-AGCACTGTGTTGGCGTACAG-3′. All mRNA measurements were normalized to the expression level of β-actin.

### Mutation Analysis of the Patient

DNA from the patient and his family members was extracted from peripheral blood lymphocytes by standard protocols. Fifteen sets of primers were designed according to the published sequence of *MUSK* (GenBank accession number # AF006464). PCR was carried out with: DNA 50 ng, dNTP 1 µM, each primer 0.5 µM, Enhancer (Tebu, Le Perray en Yvelines, France) 1X MgCl_2_ 1.5 mM, and Ampli Taq Gold 1 unit (Applied Biosystems, Courtaboeuf, France). All fragments (exons and flanking intronic regions) were amplified in a single set of conditions consisting of a touch-down protocol including a hybridization step at 65°C to 55°C (10 cycles, 1°C per cycle) followed by 25 cycles at 55°C. PCR fragments were then sequenced using the Big Dye Terminator (V3.1) Cycle Sequencing Kit (Applied Biosystems). Sequencing reactions were loaded on an ABI PRISM 3100 genetic analyzer and analyzed with Seqscape software.

### Experimental Expression of Mutated MuSK

#### Site-Directed mutagenesis

Because of the high homology between rat and human MuSK sequences, we used a pcDNA3.1 vector (Invitrogen) containing rat *musk* cDNA kindly provided by the late Prof. Werner Hoch. We added an HA-tag to the C-terminal extremity before the STOP codon by site-directed mutagenesis (GeneCust, Dudelange, Luxembourg).

The M835V mutation of human *MuSK* was reproduced in the rat *MuSK* cDNA using site-directed mutagenesis. Because of the sequence numbering difference, the homologous mutation in rat MuSK cDNA is M834V. We also introduced the mutation K608A (KA), which is known to suppress MuSK kinase activity [29].

#### *In Vivo* Expression: Electrotransfection in mouse tibialis anterior muscle

All animal studies were approved by animal use procedures adopted by INSERM. Swiss Webster mice, 5–6 weeks old, were anesthetized with 300 µl of 0.05% xylazine–1.7% ketamine in 0.9% NaCl. Seven µg of DNA containing 5 µg of the wild-type or mutated MuSK expression vectors and 2 µg of reporter plasmid were injected into the tibialis anterior muscle using a 1 ml syringe with a 27 gauge needle. Caliper electrode plates (Qbiogen, Illkirch, France) were then applied to each side of the muscle, and a series of eight electrical pulses (2 Hz, 20 ms each) was delivered with a standard square-wave electroporator (ECM 830, Qbiogen) [30]. Electrical contact was ensured by shaving and conductive gel application. The reporter construct allowed the identification of electroporated fibers, and consisted of expression vectors for a nuclear CFP (pECFPnuc, Clontech, Ozyme) [31]. Morphological analysis of the endplate was performed by confocal microscopy as described above for the patient muscle biopsy. We used both a 168 kDa and a 200 kDa neurofilament antibody.

#### *In Vitro* Expression: Stably transfected MuSK −/− muscle cells

To prepare muscle cell lines stably expressing WT- and MV-MuSK constructs, MuSK −/− muscle cells were obtained from the limbs of MuSK −/−; H-2Kb-tsA58 E18 embryos [32]. As described previously, retroviral infection of MuSK −/− myoblasts was performed following transfection of Bosc 23 cells with pBabe/puro-MuSK WT-HA and pBabe/puro-A/G MuSK-HA [10]. Infected myoblasts were selected in growth medium containing puromycin and selected clones, after verification for their ability to form myotubes and expression of MuSK, were subsequently cultured at 33°C on Matrigel-precoated dishes. Growth medium was DMEM containing 10% fetal bovine serum (FBS), 10% horse serum (HS), 0,5% chicken embryo extract (CEE) (MP Biomedicals), supplemented with 20 U/ml of recombinant mouse interferon-γ (Roche Diagnostics, France) and 50 µg/ml of gentamicin. To induce myotube formation, cultures were transferred to nonpermissive conditions by increasing the temperature to 39°C and removing FBS and IFN-γ from the medium.

#### AChR clustering assay

Myotubes were treated or not with a recombinant rat agrin (0.1 nM; R&D Systems Europe, UK). Seven hours later, they were washed with PBS, fixed with 4% paraformaldehyde in PBS for 10 minutes, washed three times with 0.1 M glycine in PBS and then blocked in PBS-3% BSA. Myotubes were then incubated for 1 h with TRITC-α-BGT (Invitrogen, France), washed three times with PBS and mounted with Vectashield DAPI (Vector Laboratories, USA). To quantitate clusters, culture dishes were observed using a 40x objective of an inverted Olympus IX70 fluorescence microscope (Olympus Europa, Hamburg, Germany). Images were recorded with a CCD camera (Princeton Cool SNAP Fx, Trenton, NJ) and processed with ImageJ software. The number of AChR clusters of more than 3 µm length in each field was divided by the number of labeled myotubes that crossed the field. Data from three independent experiments were used to calculate means ± SD.

#### Agrin-Dependent MuSK phosphorylation

293T cells, transfected with Lrp4 (kindly provided by Dr. Lin Mei), HA-tagged WT, WT/KA, MV and MV/KA MuSK expression vectors (2 µg each) via Lipofectamine 2000 (Invitrogen), were switched to DMEM without serum for three hours prior to stimulation with various concentrations of agrin (5 to 10 nM) for one hour. Cells were rinsed with ice-cold PBS and extracted in lysis buffer. Lysates were pre-cleared by centrifugation and incubated with anti-HA monoclonal antibody HA7; Sigma) overnight. Antibody was captured with protein G-Sepharose beads, which were subsequently washed four times in lysis buffer. Bound proteins were eluted from the beads with SDS sample buffer, resolved by SDS-PAGE, and transferred to Immobilon-P membranes (Millipore). Membranes were blocked in Tris-buffered saline (TBS) containing 5% skim milk and incubated with 4G10 antibody for anti-phosphotyrosine detection in TBS +3% skim milk and with HA (HA7) antibody for MuSK detection in TBS +1% skim milk. Phosphorylation was estimated with ImageJ software.

#### Dok7-Dependent MuSK phosphorylation

293T cells were transfected with HA-tagged WT or mutated MuSK and/or Dok-7 expression vectors using Fugene6 (Roche). Two days later, whole cell lysates from 293T cells were prepared with solubilization buffer (20 mM Tris-HCl, pH 7.5, 120 mM NaCl, 0.05% SDS, and 1% Triton X-100, 1 mM Na_3_VO_4_). Immunoprecipitation and immunoblotting were performed basically as described above. The following antibodies were used: anti-HA rat monoclonal antibody (3F19; Roche); anti-Dok-7 (H-77), anti-HA rabbit polyclonal antibody (Y-11) and horse-radish peroxidase (HRP)-conjugated anti-goat IgG (Santa Cruz Biotechnology); anti-phosphotyrosine (4G10; Upstate Biotechnology); HRP-conjugated anti-mouse and rat IgG (GE Healthcare). Relative signal intensity of MuSK proteins was measured using NIH Image (v. 1.63) software, and the densitometric measurements of tyrosine phosphorylation levels for MuSK were normalized to the total amount of MuSK proteins.

## Results

### Clinical Data

A 12-year-old boy presented with congenital bilateral ptosis and slowly progressive muscle weakness, particularly in the lower limbs, which had been observed since the age of 4. He had no sucking problems during infancy and started to walk at 14 months. A history of nocturnal sleep apnea during childhood was mentioned by the parents. There was aggravation of symptoms following infections or exposure to cold without fluctuation of weakness during the day. There were neither swallowing problems nor any crisis requiring hospitalization. His parents were cousins from north-western province of Qazvin in Iran. He had a healthy younger sister. There was no history of similar diseases in the family.

Neurologic examination revealed severe bilateral symmetric ptosis and upward gaze palsy, without involvement of the orbicularis oculi. Atrophy of distal lower limb muscles and the tongue was noted. There were no fasciculations, scoliosis, or joint contractures. Bulbar and masticatory muscles were intact, while exertional dyspnea and mild axial muscle weakness were noted. Muscle weakness in all four limbs was more marked proximally and there was a waddling gait as well as positive Gowers’ sign. Based on the quantitative myasthenia gravis score [33], proximal upper limbs showed moderate clinical fatigability and lower limb proximal muscles revealed mild clinical fatigability. Deep tendon reflexes and sensory data were normal.

The anticholinesterase test with ambenonium chloride (Mytelase) or edrophonium chloride (Tensilon) showed no dramatic positive response. Serum creatine kinase concentration was 53 U/L and anti-nAChR antibodies were not present. Electrocardiography and echocardiography showed no significant abnormality, while vital capacity on spirometry was 68% of the normal value.

Sensory and motor nerve conduction velocities were normal. On electromyographic examination, motor units showed a myopathic pattern with low amplitude short duration polyphasic potentials detected in several muscles. No significant decremental response was noted on 3 Hz repetitive stimulation of forearm and spinal muscles.

Computerized tomography of limb muscles showed diffuse, global amyotrophy, predominantly in the lower limbs without any density abnormalities in the observed muscle masses.

Treatment with cholinesterase inhibitors (pyridostigmine) was not successful. The patient underwent muscle biopsy and blood samples were taken for genetic studies. Six years after his first visit (at 18 years of age), he lost ambulation and could walk only with help. The patient had one episode of generalized tonic-clonic seizure at the age of 18 and two successive (within four hours) seizures at 19 years old, which were then controlled by carbamazepine. Three months later, he developed a severe pulmonary infection and died at a local intensive care unit within one week.

### Genetic Analysis

**Figure 1:**
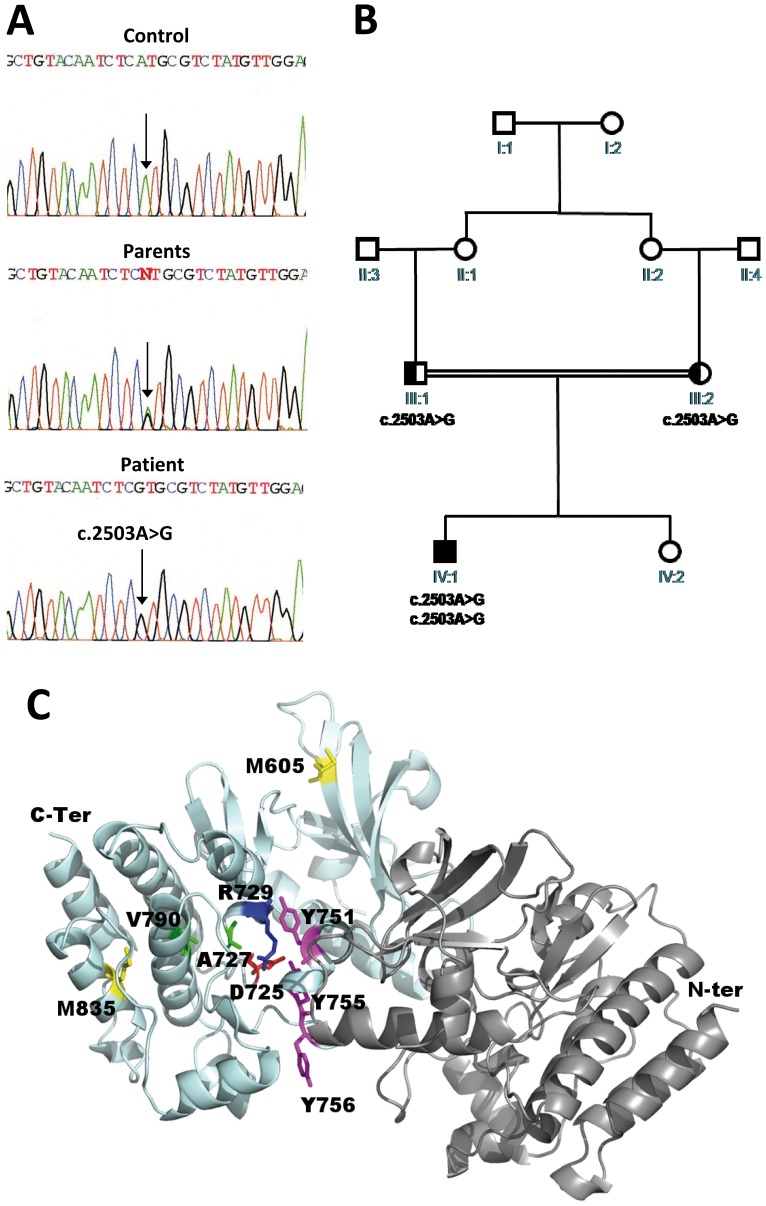
Identification of the homozygous c.2503A>G in *MUSK*, hereditary transmission and localization of the mutation in a three-dimensional model of the intracellular part of MuSK. (**A**) Sequence chromatograms from a normal individual (control), affected proband IV: 1 and his parents (parents) are shown. (**B**) Pedigree of the Iranian family. The proband is indicated by an arrow. The expected nucleotide change c.2503A>G transmitted in this consanguineous Iranian family is indicated below the symbols when determined. (**C**) 3D model of the cytoplasmic kinase domain dimer of human MuSK built by homology modeling based on the structure of rat MuSK (PDB code: 1LUF) [11] superimposed on the structure of the Insulin-like Growth factor 1 (PDB code 3D94), as proposed recently [34]. The model is shown in cartoon representation; also represented in sticks are the catalytic residues (Tyrosines 751, 755 and 756, Aspartate 725 and Arginine 729) and residues whose mutations are associated with myasthenic syndromes A727/M605, V790 and M835). N-terminus, C-terminus and a loop not resolved in the structure are also indicated.

Initial screening for mutations in the epsilon subunit of AChR and rapsyn genes was negative. Based on the histopathologic findings and the previous report of a family with a mutation in *MUSK*, coding sequences of *MUSK* and their flanking regions were sequenced. A homozygous mutation consisting of an A to G transition at nucleotide 2503 of the coding sequence in exon 15: c.2503A>G was identified (Figure 1 A). This mutation leads to the missense substitution p.Met835Val (M835V). Analysis of family members showed that the mutation was transmitted by each heterozygous parent (Figure 1 A, B). None of the 200 control chromosomes from a sample of the Iranian population, nor the 200 French control chromosomes harbored this mutation.

### Localization of the Mutation on a 3D Model of the Cytoplasmic Domain of MuSK

The mutation is located near the C-terminal of MuSK and leads to a methionine to valine substitution at codon 835. A model of the human cytoplasmic kinase domain dimer of human MuSK was built by homology modeling based on the structure of rat MuSK (PDB code: 1LUF) [11] superimposed on the structure of the Insulin-like Growth factor 1 (PDB code 3D94), as proposed recently [34]. Inspection of the model reveals that the residue M835 is not exposed to the solvent, and is close to residues already known to be involved in myasthenic syndromes (V790, and A727) [23], [27], but not in direct proximity to the catalytic residues or interface of dimerisation (Figure 1 C).

### Muscle Biopsy Study

Whole fibers of the biopsied deltoid muscle were studied by coimmunolabeling of nAChR and neurofilaments then observed by confocal microscopy. Major alterations were observed in different compartments of the NMJ.

**Figure 2:**
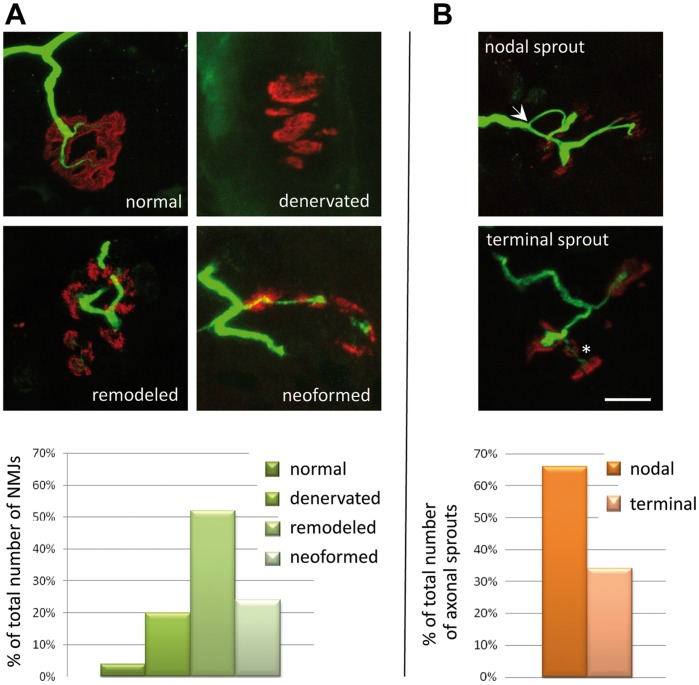
Morphological study of the patient’s biopsied muscle. (**A**) Whole-mount preparations stained with α-bungarotoxin for nAChR in red and with an anti-neurofilament (NF) antibody for axons in green. In the normal NMJ, the axonal branch typically ends as a fork innervating a well-defined synaptic structure. In denervated NMJ, axons are absent. In some cases, axonal sprouts induce aggregation of nAChR forming ectopic junctions as a rosary. The histogram shows the classification of the NMJs observed in the patient muscle-biopsy specimen into four categories (expressed as percentage of the 25 NMJs examined). (**B**) In remodeled NMJs, nodal (arrow) or terminal (asterisk) axonal sprouts reinnervate synaptic gutters. The histogram shows the classification of the sprouting profiles observed into two categories (expressed as a percentage of the 16 NMJs exhibiting sprouts identified amongst the 25 NMJs analyzed). Scale bar = 10 µm and applies to all prints in A and B.

**Figure 3:**
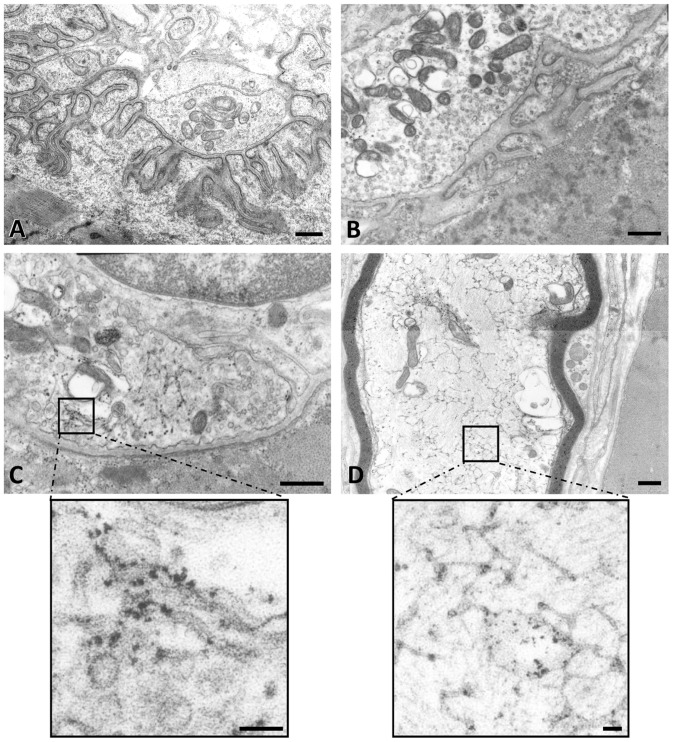
Electron microscopy in control and patient. (**A**) Control endplate Patient’s endplates: (**B**) reinnervated endplate shows decreased and enlarged post-synaptic folds, a vanished basal lamina, and absence of active zones. (**C**) Ectopic NMJ is characterized by the total absence of subneural folds indicating that they are at an early stage of synaptogenesis. (**D**) An axon with a smooth endoplasmic reticulum (SER) network, a sign of impaired axonal flow. Zooms show aggregation of unidentified proteins on SER networks. Scale bars represent 500 nm for the four low-magnification prints and 3 µm for both zooms.

In the presynaptic compartment, unlike a normal nerve terminal which ends by dividing into several branches, the terminal axon at best bifurcated in contacting small scattered synaptic cups (Figure 2 A). From this primary innervation axonal sprouts had emerged and contacted nAChR cups, so increasing the synaptic territory. 66% of the axonal sprouts were nodal and 34% terminal or ultraterminal (Figure 2 B). In ultrastructural analysis, myelinated axons exhibited a network of smooth endoplasmic reticulum that could represent a sign of perturbed axonal flow (Figure 3 C&D) [35].

The basal lamina as seen by electron microscopy was undefined with an unorganized granular substance at the synaptic cleft or, sometimes, a duplication of its structure in enlarged subneural folds (Figure 3 B).

In the post-synaptic compartment, 25 NMJs were analyzed and classified into categories according to the structural state of this compartment: only 1 (4%) had a normal shape, 5 (20%) were “denervated” with fragmented and dispersed synaptic gutters with evanescent borders and absence of nerve terminals, 13 (52%) “remodeled” with partially denervated and reinnervating profiles, and 6 (24%) showing “neoformed”synaptic cups (Figure 2 A).

In the ultrastructural study, these neoformed NMJs were very immature and characterized by total absence of subneural folds (Figure 3 C). In the remodeled NMJs, the subneural folds were enlarged and markedly decreased in both number and length (Figure 3 B).

#### Immunocytochemistry of transverse cryostat sections

**Figure 4:**
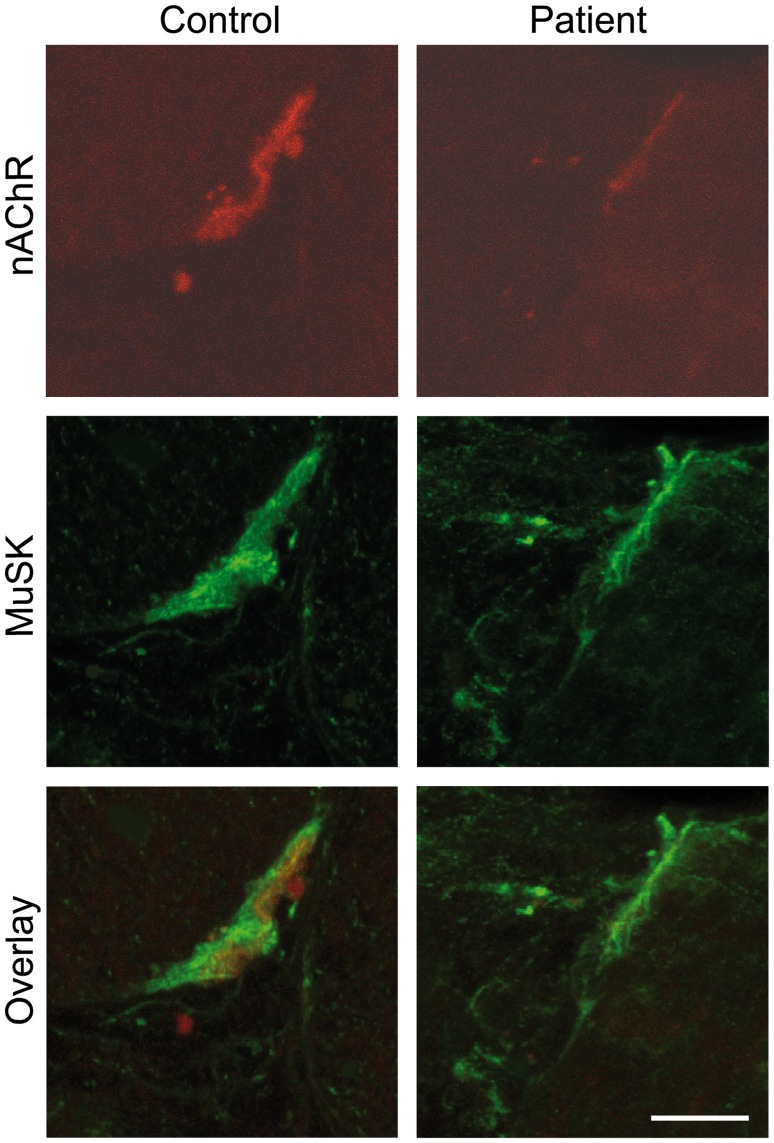
Immunocytochemistry of MuSK on cryostat sections. In the control, nAChR and MuSK are strongly expressed and perfectly colocalized. In the patient, nAChR expression is reduced whereas MuSK expression remains normal and extends slightly in the perisynaptic area. The scale bar represents 10 µm and applies to the six prints.

In control muscle, nAChR and MuSK were colocalized (Figure 4). In the patient muscle MuSK labeling was not strictly colocalized with nAChR since it extended beyond the NMJ area. In addition, nAChR staining was reduced in comparison with the control while no obvious difference of MuSK staining could be observed.

**Figure 5:**
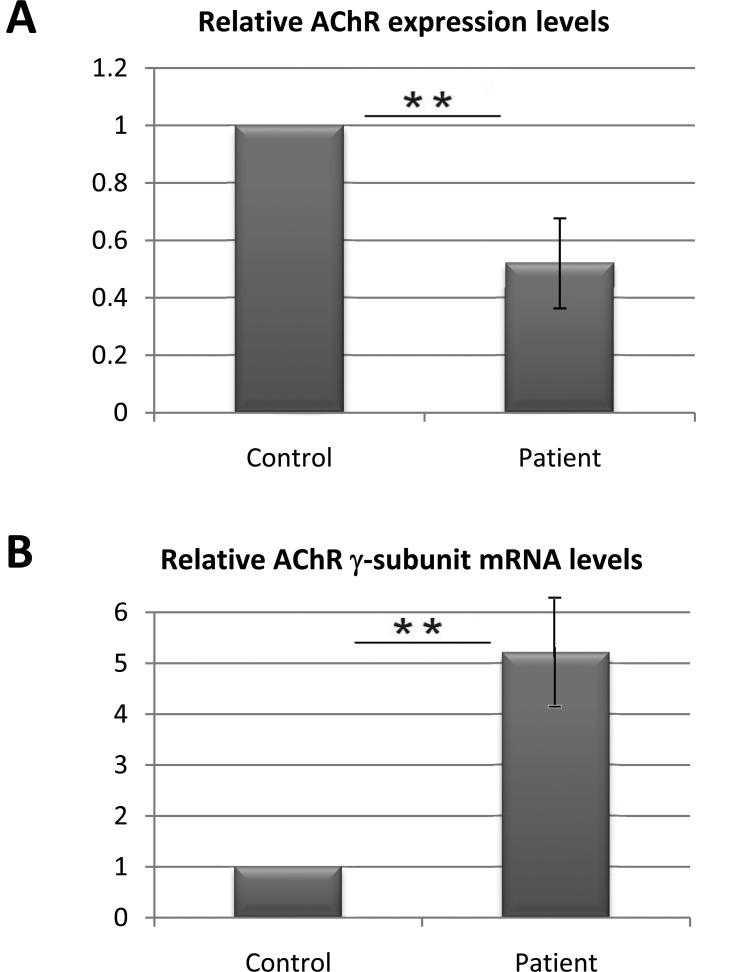
Quantification of nAChRs in patient’s biopsied muscle. (**A**) nAChR expression in MuSK mutated muscle from the patient was decreased by about 50% compared with biopsied control muscle. (**B**) The nAChR γ-subunit mRNA level, which is synonymous with denervation, was measured in the extrasynaptic zone in patient and was increased by 5-fold compared with the control. The mean value of control AChR (A) or AChR γ-subunit mRNA levels were defined as 1 in arbitrary units. Error bars indicate mean±SD (**, P<0.01, Student *t* test).

As there was a decreased concentration of nAChR, we measured the total area of the synaptic gutters of each NMJ and the mean fluorescent intensity of the α-bungarotoxin staining in patient versus control confocal images and noted a ∼50% decrease in nAChR in the patient versus control (Figure 5 A). Using quantitative real-time PCR (qRT-PCR), we quantified the expression levels of nAChR γ-subunit mRNA in extrasynaptic sections. We found an increased expression of the γ-subunit mRNA compared with the control (Figure 5 B), which reflects the functional denervation state of muscle.

### Experimental Expression of the Mutation

In order to evaluate the pathogenicity of the mutation of methionine 835 of human *MuSK* into a valine, this mutation was reproduced in a rat *MuSK* cDNA using site-directed mutagenesis. Because of the sequence numbering difference, the homologous mutation in rat MuSK cDNA is M834V. For simplification, we will use the symbol MV to describe the M834V mutation.

#### *In Vivo* Expression: Electroporation of MV-MuSK in mouse muscles

**Figure 6:**
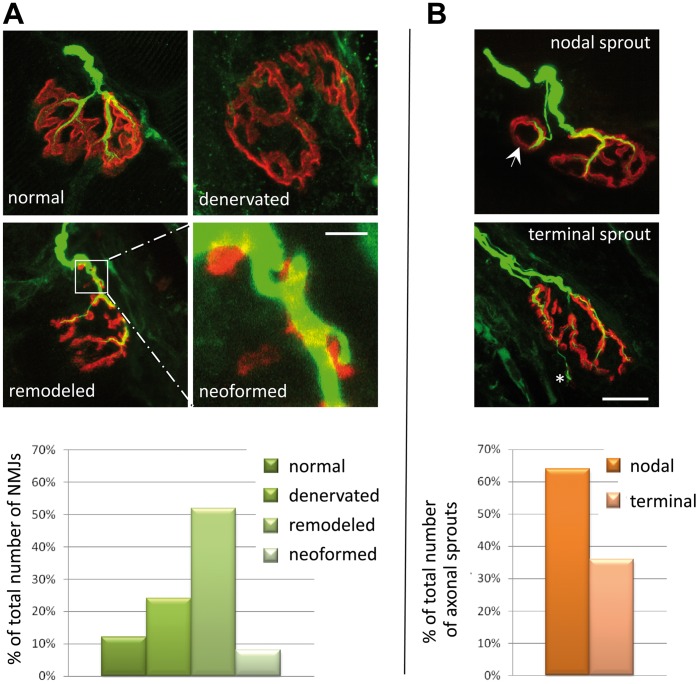
Morphological study of electroporated mice. (**A**). Denervated junctions were fragmented and dispersed without terminal axons. Remodeled junctions were partially reinnervated and showed nodal sprouts forming more or less mature ectopic junctions (**B**). The arrow shows a mature ectopic junction with well-defined borders synonymous with subneural fold formation. The asterisk and the zoom show the first steps of ectopic junction formation characterized by very simplified AChR clusters induced by emergent nodal sprouts or axonal varicosity. Histograms show the distribution of different types of NMJs and sprouting in MV MuSK electroporated mice. The scale bar represents 10 µm and applies to all prints except to the blowup where the scale bar represents 2.5 µm.

To evaluate the effect of the MV mutation in MuSK, wild-type (WT) or mutant (MV) MuSK expression vectors were electroporated in mouse tibialis anterior muscle. NMJ morphology was studied by confocal microscopy following immunolabeling for nAChR and neurofilaments. NMJs were then classified into several morphological categories according to the NMJ categorization used for the patient. Among the NMJs counted, 12% were normal, 24% denervated, 56% remodeled and 8% neoformed (Figure 6 A). As in the patient biopsy specimens, denervated NMJs exhibited fragmented gutters with evanescent borders and absence of nerve terminals (Figure 6 A).

In a remodeled NMJ, the terminal axon showed reduced nerve ramification which contacted some synaptic gutters. Furthermore, a nodal sprout budded from this axon contacting an ectopic ring-shaped junction with well-defined borders synonymous with maturation (Figure 6 B). We observed the first stage of ectopic NMJ formation. These NMJs are small clusters of AChRs induced by short nascent axonal nodal sprouts (Figure 6A). The breakdown of axonal sprouts was similar to what was seen in patient NMJs: 64% nodal and 36% terminal or ultraterminal (Figure 6 C). We also measured the total area of the synaptic gutters of each NMJ and the mean fluorescent intensity of the α-bungarotoxin staining in MV- versus WT- MuSK electroporated animals and noted a ∼50% decrease in nAChR per NMJ with MV versus WT MuSK.

#### Effect of the MV-MuSK mutation on AChR clustering

To test whether the mutation could affect nAChR aggregation, MuSK −/− cells expressing similar levels of WT- or MV-MuSK were used. MuSK −/− cells were used as control. Differentiated myotubes (2–6 nuclei) were treated or not with agrin (0.1 nM).

**Figure 7:**
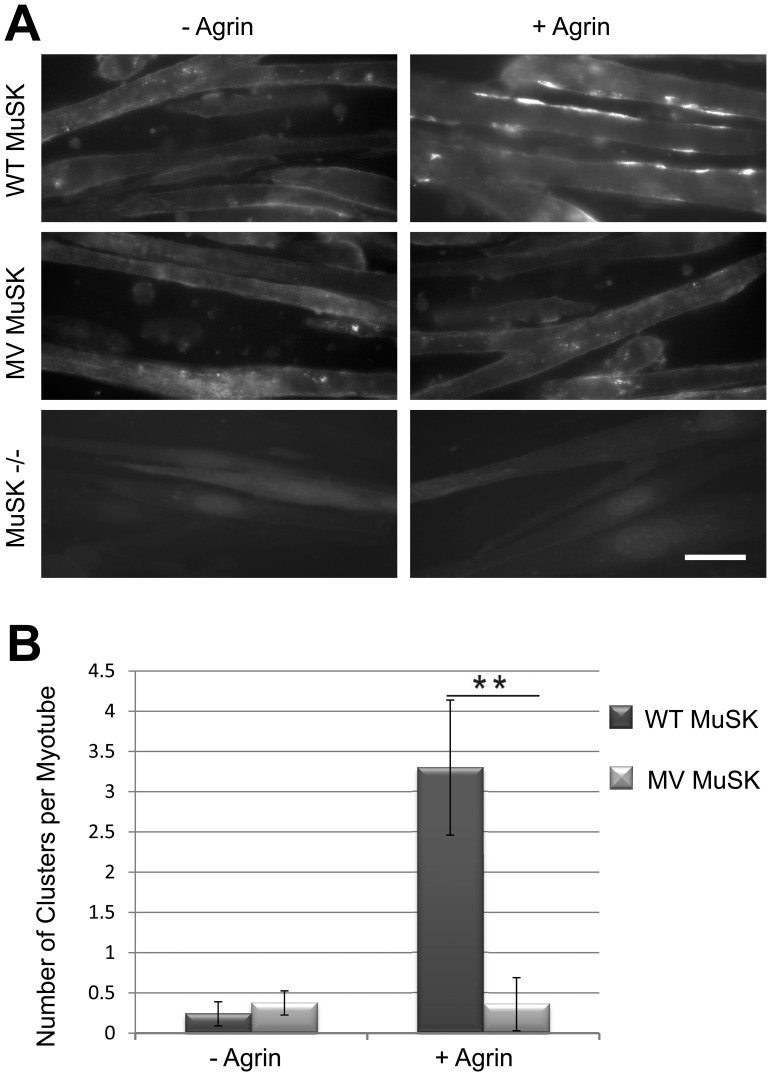
Effect of the MV MuSK mutation on AChR aggregation. (**A**) MuSK −/− cells failed to aggregate nAChR with or with agrin treatment. We observed some aneural nAChR clusters in both WT and MV MuSK cells. After agrin treatment, nAChR clusters of more than 3 µm length appeared in WT MuSK cells while they remained almost undetectable in MV MuSK cells. Scale bar = 20 µm. (**B**) Histogram showing the number of nAChR aggregates of more than 3 µm per myotube that crossed the field from 3 independent experiments (20 fields in each Petri dish were randomly counted for each set of experimental conditions). Error bars indicate mean±SD (**, P<0.01, Student *t* test).

Without agrin stimulation, nAChR aggregation was absent in MV and WT myotubes and virtually no clusters were observed. After agrin treatment nAChR aggregation became evident in WT myotubes and reached ∼3 nAChR clusters per myotube whereas it remained unchanged, i.e. close to the baseline in MV myotubes (Figure 7).

#### Evaluation of agrin-dependent MuSK phosphorylation

**Figure 8:**
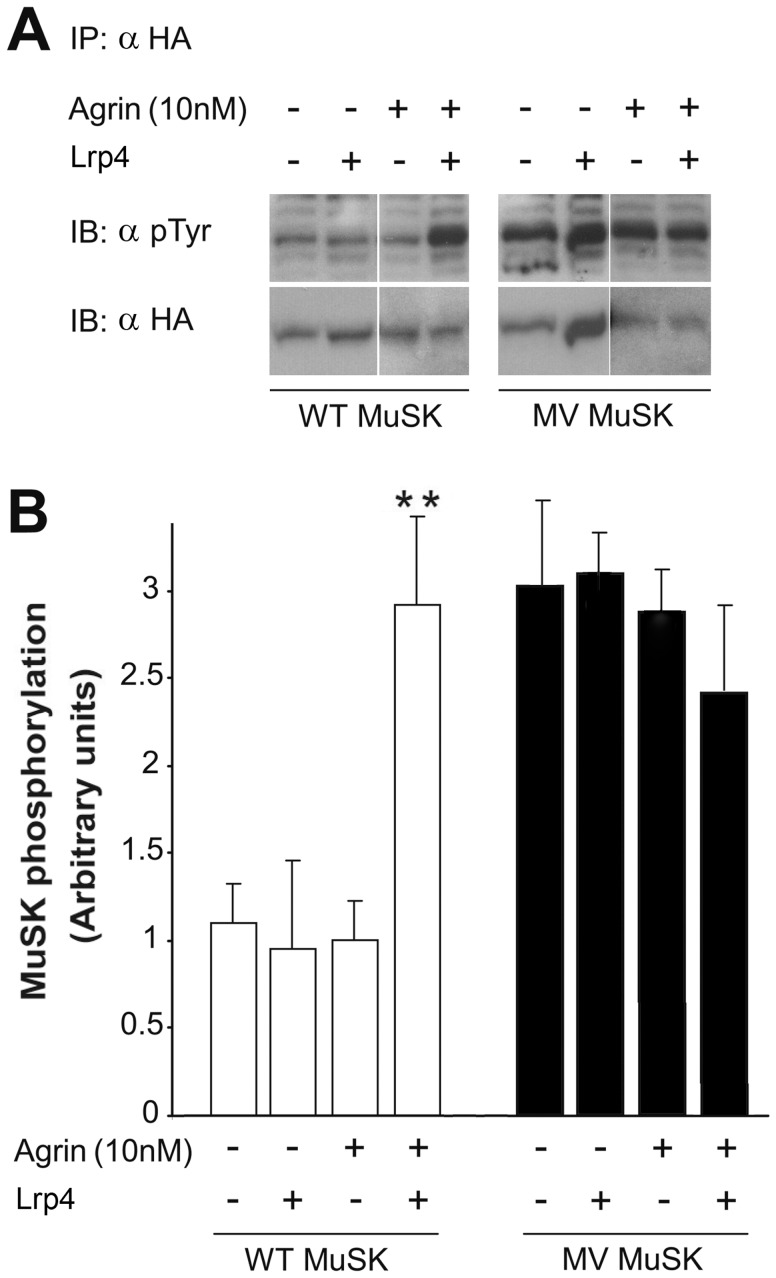
Effect of the mutation on agrin-dependent MuSK phosphorylation. HEK 293 cells expressing both Lrp4 and MuSK were incubated with recombinant rat agrin. (**A**) HA-MuSK was immunoprecipitated (IP) with an anti-HA antibody (α HA) and immunoblots (IB) for MuSK (α HA) and phosphorylated MuSK (α pTyr) were performed. A representative experiment out of three realized in duplicate is shown. MuSK amount and phosphorylation were then estimated with ImageJ software. (**B**) Phosphorylation is expressed as n-fold activation as compared to WT MuSK without Lrp4 and agrin. Error bars indicate mean±SD (n = 3; **, P<0.01, paired *t* test). Comparison between MV and WT MuSK shows that while WT MuSK needs the presence of both agrin and Lrp4 to be phosphorylated, MV MuSK is already phosphorylated even in the absence of Lrp4 and agrin.

**Figure 9:**
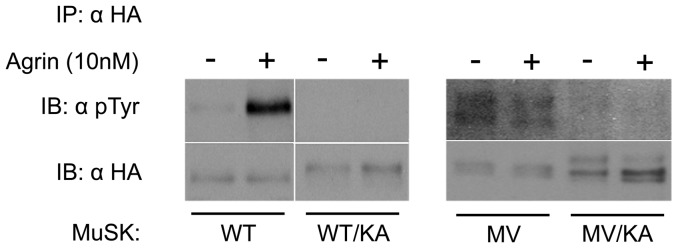
Effect of the use of a dead-kinase MuSK on MV and WT MuSK phosphorylation. HEK 293 cells expressing both Lrp4 and MuSK were incubated with recombinant rat agrin. HA-MuSK was immunoprecipitated (IP) with an anti-HA antibody (α HA) and immunoblots (IB) for MuSK (α HA) and phosphorylated MuSK (α pTyr) were performed. A representative experiment out of three realized in duplicate is shown. As expected the introduction of the dead-kinase mutation in WT MuSK (WT/KA) abolishes the phosphorylation observed in the presence of both Lrp4 and agrin. The introduction of the kinase-dead mutation together with the MV mutation (MV/KA) abolishes the spontaneous phosphorylation of MV MuSK (MV) seen in the absence of Lrp4 and agrin.

To investigate if MuSK activation was impaired by the mutation, MuSK phosphorylation was measured in 293T cells transfected with WT- or MV-MuSK constructs in combination or not with Lrp4. The cells were further treated or not with agrin (10 nM). MuSK was immunoprecipitated from the cell lysate with an anti-HA antibody, and visualized on western blots with the same antibody as well as with an anti-phosphotyrosine antibody (Figure 8). As expected, Lrp4 was required for WT-MuSK phosphorylation in response to agrin. However, it had no effect on MV-MuSK phosphorylation. Indeed, the MV-MuSK mutant, either alone or in combination with Lrp4, was already strongly phosphorylated in the absence of agrin, and agrin treatment did not further stimulate its phosphorylation. In other words the MV-MuSK mutant was always more phosphorylated (3 times more) than WT MuSK, except in the presence of both Lrp4 and agrin where WT-MuSK reached the same level of activation as MV-MuSK mutant. This suggests that the mutation impairs the activation of MuSK phosphorylation by agrin by conferring to MuSK elevated basal levels of phosphorylation. To test whether this unexpected phosphorylation of MV MuSK was due to the phosphorylation of normally unexposed tyrosine residues, unmasked by a change in the conformation of MV MuSK, by another cellular kinase, we expressed a kinase-dead MV-MuSK. We concluded that this was not the case since phosphorylation of MV MuSK was completely abolished in the context of an inactive MuSK kinase activity (Figure 9).

#### Evaluation of Dok7-Dependent MuSK phosphorylation

**Figure 10:**
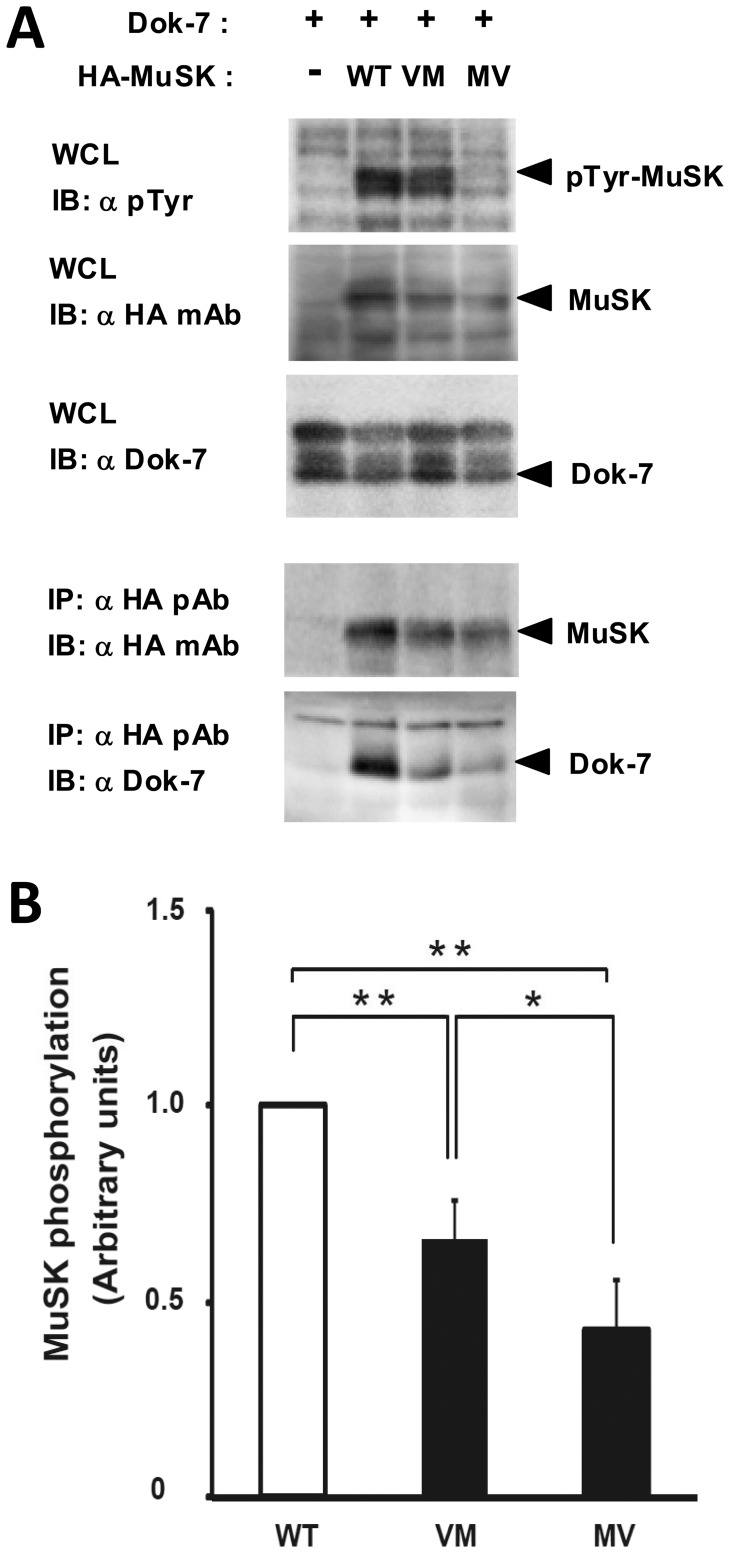
Effects of the mutations on Dok-7-dependent MuSK phosphorylation and its binding to Dok-7. HA-MuSK (WT, VM, or MV) and/or WT-Dok-7 proteins were exogenously expressed in 293T cells and the whole cell lysates (WCLs) were subjected to immunoprecipitation (IP) of MuSK proteins with anti-HA rabbit polyclonal antibody (α HA pAb) and immunoblotting (IB) with anti-HA rat monoclonal antibody (α HA mAb), anti-Dok-7 antibody (α Dok-7), or anti-phosphotyrosine antibody (α pTyr). (**A**) Representative data from 3 independent experiments are shown. (**B**) Ratios of the tyrosine phosphorylation of MuSK to total amount of MuSK were quantified and shown. The mean value of Dok-7-dependent MuSK (WT) phosphorylation levels was defined as 1 in arbitrary units. Error bars indicate mean±SD (n = 3; *, P<0.05; **, P<0.01, paired *t* test).

Since phosphorylation of MuSK is critically influenced by its co-activator Dok7 [15], the agrin-independent phosphorylation of MuSK was evaluated after the co-transfection of WT or MV MuSK constructs with Dok-7 in 293T cells. V790M, another *MUSK* mutation was also studied [23]. Immunoblotting of whole cell lysates (WCLs) with anti-HA antibody (to reveal MuSK), anti-Dok7 antibody, and anti-phosphotyrosine antibody showed that the levels of expression of WT, VM and MV MuSKs were similar as was that of Dok7 in all lanes. However, immunoblotting with an anti-phosphotyrosine antibody revealed that MV-MuSK was significantly less activated by Dok7 than WT-MuSK, and even less than V790M-MuSK (Figure 10 A, B).

Immunoprecipitation of WCLs with anti-HA antibody, followed by immunoblotting with the same antibody showed that WT, VM and MV MuSKs were expressed at similar levels. In addition, immunoblotting of the immunoprecipitate with a Dok7 antibody showed that Dok7 was well co-immunoprecipated with WT MuSK. Conversely, Dok7 did not co-immunoprecipitate efficiently with MV-MuSK, and even less than with V790M-MuSK. This suggests that the binding of Dok-7 to MuSK was impaired for both V790M-MuSK and MV-MuSK mutations (Figure 10 A).

## Discussion

We report a novel mutation in *MUSK* leading to a CMS. We formerly described two CMS siblings with a frameshift and a missense mutation in MuSK (c.220insC and V790M) [23]. Since then, only two other families have been described with CMS due to MuSK mutations [26], [27]. The one reported in this paper is a homozygous missense mutation M835V located in the kinase domain, like the previously reported mutations V790M [26], M605I and A727V [27].

The patient had a progressively deteriorating course starting from infancy with some worsening in cold weather and during infections, and finally died of a pulmonary infection, while the previous MuSK patient (bearing c.220insC with V790M mutations) showed, apart from her infancy, a more favorable course during the first two decades of life and at the present time is only suffering from ptosis and upper limb weakness. However, it should be noted that our Iranian patient was never treated by the combination of pyridostigmine and diaminopyridine (DAP), which was very efficacious for our first case [23] and mildly beneficial for the Sudanese siblings [26]. In contrast to many myasthenic patients, our patient did not show daytime fluctuation of symptoms with worsening in the evening, bulbar symptoms or frank decremental response on electromyography. Tongue atrophy, which is a characteristic sign in the anti-MuSK antibody positive type of autoimmune myasthenia [36], was a remarkable finding in our patient and has also been reported in two CMS patients with Dok-7 mutations [37].

MuSK is known to be a key component in orchestrating both formation and maintenance of NMJs. The patient’s biopsy exhibited high plasticity of the NMJs with about 70% of denervated or remodeled NMJs. It also showed a 50% decrease of the average number of nAChR (-50%) and a compensatory increase of axonal sprouts, as was observed in mouse, where MuSK perturbation in adult muscle by RNA interference also induced sprouting of presynaptic nerve terminals [18].

Two types of sprouting can be distinguished in the biopsies: terminal and nodal, with a major predominance of the latter. It is assumed that **terminal sprouts** may be due to NMJ local factors related to insulin-like growth factors (IGFs) [38]. Muscle IGFs could play a role in intramuscular nerve sprouting and are probably signaling factors from inactivated muscle to promote local restorative reactions [39], [40]. **Nodal sprouts** appear far from the synaptic cleft and are therefore unlikely to be under direct influence of NMJ local factors. We assume that their formation is partly controlled by factors released by muscle or Schwann cells. Such factors could be modified then transported by the retrograde axonal flow. Muscle-derived retrograde signals might also be passed to motor neuron terminals via terminal Schwann cells, which are essential components for growth, maturation, and maintenance of the NMJ [40], [41]. The hypothesis of disturbed axonal flows is particularly supported by the presence of a smooth endoplasmic reticulum network in the patient’s axons. This smooth endoplasmic reticulum network has already been described in frontal biopsy specimens from patients with Alzheimer's disease [42] and in motor axon from Trembler mice [35] and can be attributed to disturbances of axoplasmic flow.

Evidence that the M835V mutation is responsible for high NMJ remodeling capacity, decreased number of nAChR and both nodal and terminal compensatory innervation is provided by reproducing these same results in electroporated mouse muscle. The fact that the mutated MuSK was expressed in the presence of endogenous MuSK could suggest that the mutated MuSK had a dominant negative effect on wild-type MuSK. This would be in contradistinction with the recessive expression of the mutation in human. The most probable explanation is that, owing to the electroporation efficiency, the number of mutated MuSK molecules sufficiently exceeded that of endogenous MuSK to displace it from all its cellular partners and thus, that only the mutated MuSK was functional at the NMJ. In other words, the fact that elevated levels of mutated MuSK generated the same effect as in the patient likely shows that the phenotype is not attributable to reduced levels of MuSK, but rather to an altered ability of the mutated MuSK to perform its physiological function.

An interesting observation was that agrin had almost no effect on the ability of the MV MuSK mutant to induce nAChR aggregation, while a twofold reduction of the clusters was observed in agrin-treated muscle cells expressing either MuSK V789M or MuSK M605I as compared with cells expressing MuSK WT [23], [27].

To address this point, MuSK phosphorylation was measured in heterologous cells in the absence of agrin and we found that MV MuSK was already phosphorylated and that agrin treatment did not significantly increase its phosphorylation. This “spontaneous” phosphorylation was not due to phosphorylation of other residues than those phosphorylated in WT-MuSK since introducing the kinase-dead mutation K608A completely abolished phosphorylation both in WT and MV-MuSK. It is generally accepted that the activation of MuSK is linked to its dimerization [43]. Consistently, the expression of the MuSKneuTMuSK homodimer in the extrasynaptic zone of a muscle induces the formation of AChR aggregates [44]. Whether or not conformational changes of MuSK, which are induced by the mutation - and discussed below - could lead to such spontaneous homodimerization of MuSK remains speculative, especially *in vivo*.

As opposed to other RTKs which are only activated by their ligand, MuSK phosphorylation is known to be also increased by Dok-7, at least when overexpressed [15], [34], [45]. We therefore asked if the induction of MuSK phosphorylation by Dok-7 was impaired by the mutation. Interestingly, MV MuSK phosphorylation in the presence of Dok7 was 60% lower than with WT MuSK. MuSK-Dok-7 co-immunoprecipitation experiments further showed that this could be due to reduced binding of Dok-7 to the mutated MuSK. It therefore appears that mutations in *DOK7* or *MUSK* leading to a CMS can impair at various levels MuSK phosphorylation and MuSK binding to Dok-7 [24], [27], [46]. This impairment was striking for the present homozygous M835V *MUSK* mutation, more than for our previously described mutation (*MUSK* V790M) [23]. Being faced with the end-result in the patient, which is a twofold decrease of AChR number at the NMJ, knowing which combination of the above mechanisms (agrin/LRP4 and Dok-7 interaction with mutated MuSK) is actually taking place in the patient remains a question.

According to the 3D model of MuSK structure [11], the M835 residue is neither exposed to the solvent nor proximal to the catalytic residues. The effect of the mutation of this residue on MuSK phosphorylation is therefore likely to be allosteric rather than steric. An allosteric effect may be related to a loss of stability of the protein or a modification of its structure; however the M to V mutation might not be strong enough to induce such a drastic change at the molecular level because Methionine and Valine are both small hydrophobic residues. The mutation could also alter the conformational equilibrium associated with MuSK function. A similar explanation has been suggested in the case of the nAChR [47], [48] following the observation that myasthenic mutations are not randomly distributed in the structure but rather clustered in zones that can control the activation of the receptor. Interestingly, the M835 residue is in the vicinity of V790 and A727, which are also associated with myasthenic syndromes; this proximity might be interpreted as an argument in favor of an allosteric effect modifying the conformational equilibrium. We can therefore speculate that the M835V mutation induces a change in function that counteracts agrin- and Dok-7-dependent typical activation of MuSK and that, *in fine*, is responsible for the patient’s myasthenic syndrome.
