# Correction: A Mutation Causes MuSK Reduced Sensitivity to Agrin and Congenital Myasthenia

**DOI:** 10.1371/annotation/3ff2b918-c83c-4c6f-a2e2-4d91294ec92f

**Published:** 2013-09-23

**Authors:** Asma Ben Ammar, Payam Soltanzadeh, Stéphanie Bauché, Pascale Richard, Evelyne Goillot, Ruth Herbst, Karen Gaudon, Caroline Huzé, Laurent Schaeffer, Yuji Yamanashi, Osamu Higuchi, Antoine Taly, Jeanine Koenig, Jean-Paul Leroy, Fayçal Hentati, Hossein Najmabadi, Kimia Kahrizi, Manouchehr Ilkhani, Michel Fardeau, Bruno Eymard, Daniel Hantaï

The following information was missing from the funding section: Austrian Science Fund (P21667-B09). 

